# The Delta-Specific Opioid Glycopeptide BBI-11008: CNS Penetration and Behavioral Analysis in a Preclinical Model of Levodopa-Induced Dyskinesia

**DOI:** 10.3390/ijms22010020

**Published:** 2020-12-22

**Authors:** Mitchell J. Bartlett, Omar S. Mabrouk, Lajos Szabò, Andrew J. Flores, Kate L. Parent, Jean M. Bidlack, Michael L. Heien, Robert T. Kennedy, Robin Polt, Scott J. Sherman, Torsten Falk

**Affiliations:** 1 Department of Neurology, College of Medicine, University of Arizona, Tucson, AZ 85724, USA; mbartlet@arizona.edu (M.J.B.); ssherman@neurology.arizona.edu (S.J.S.); 2 Department of Chemistry, University of Michigan, Ann Arbor, MI 48109, USA; omarmabrouk@hotmail.com (O.S.M.); rtkenn@umich.edu (R.T.K.); 3 Department of Chemistry & Biochemistry, University of Arizona, Tucson, AZ 85721, USA; lzszabo@arizona.edu (L.S.); kateparent21@gmail.com (K.L.P.); mheien@arizona.edu (M.L.H.); polt@arizona.edu (R.P.); 4 Graduate Interdisciplinary Program in Physiological Sciences, University of Arizona, Tucson, AZ 85724, USA; ajf1@email.arizona.edu; 5 Department of Pharmacology and Physiology, University of Rochester School of Medicine and Dentistry, Rochester, NY 14642, USA; jean_bidlack@urmc.rochester.edu; 6 Department of Pharmacology, University of Arizona, Tucson, AZ 85724, USA

**Keywords:** L-DOPA, Parkinson’s disease, basal ganglia

## Abstract

In previous work we evaluated an opioid glycopeptide with mixed μ/δ-opioid receptor agonism that was a congener of leu-enkephalin, MMP-2200. The glycopeptide analogue showed penetration of the blood–brain barrier (BBB) after systemic administration to rats, as well as profound central effects in models of Parkinson’s disease (PD) and levodopa (L-DOPA)-induced dyskinesia (LID). In the present study, we tested the glycopeptide BBI-11008 with selective δ-opioid receptor agonism, an analogue of deltorphin, a peptide secreted from the skin of frogs (genus *Phyllomedusa*). We tested BBI-11008 for BBB-penetration after intraperitoneal (*i.p.*) injection and evaluated effects in LID rats. BBI-11008 (10 mg/kg) demonstrated good CNS-penetrance as shown by microdialysis and mass spectrometric analysis, with peak concentration levels of 150 pM in the striatum. While BBI-11008 at both 10 and 20 mg/kg produced no effect on levodopa-induced limb, axial and oral (LAO) abnormal involuntary movements (AIMs), it reduced the levodopa-induced locomotor AIMs by 50% after systemic injection. The *N*-methyl-D-aspartate receptor antagonist MK-801 reduced levodopa-induced LAO AIMs, but worsened PD symptoms in this model. Co-administration of MMP-2200 had been shown prior to block the MK-801-induced pro-Parkinsonian activity. Interestingly, BBI-11008 was not able to block the pro-Parkinsonian effect of MK-801 in the LID model, further indicating that a balance of mu- and delta-opioid agonism is required for this modulation. In summary, this study illustrates another example of meaningful BBB-penetration of a glycopeptide analogue of a peptide to achieve a central behavioral effect, providing additional evidence for the glycosylation technique as a method to harness therapeutic potential of peptides.

## 1. Introduction

The opioid neurotransmission system is prominent in the vertebrate basal ganglia. Endogenous opioid peptides and their precursors are expressed at high levels within the basal ganglia, where they are modulators of dopamine (DA) and glutamate neurotransmission [1]. This makes the opioid system of interest for Parkinson’s disease (PD), where death of dopaminergic neurons in the *substantia nigra* and loss of striatal DA are central to the hypokinetic movement symptoms. This is also true for a disorder manifested by abnormal involuntary movements (AIMs) that develops after prolonged DA replacement therapy with levodopa (L-DOPA) in individuals with PD, L-DOPA-induced dyskinesia (LID) [2]. Furthermore, all three opioid receptor subtypes—δ (DOR), μ (MOR) and κ (KOR)—are expressed with a high density in the striatum [1]. It is of particular importance that the medium spiny neurons (MSNs), which constitute the principal outflow pathway of the striatum, are functionally segregated according to their differential expression of opioid peptide co-transmitters. The MSNs use gamma-aminobutyric acid (GABA) as their main neurotransmitter and project from the striatum to the pallidum either directly or indirectly though the subthalamic nucleus. The MSNs of the *direct pathway*, which facilitate movement, express the opioid peptide precursor preproenkephalin-A (PPE-A, Penk), while the neurons that give rise to the *indirect pathway*, which inhibit movement, express the opioid precursor preproenkephalin-B (PPE-B, Pdyn). The initial condition of PD and the subsequent development of LID are both associated with alterations in mRNA and peptide levels of these opioid precursors [1,3]. Thus, the regulation of opioid peptides within the basal ganglia is closely associated with the regulation of DA transmission [4,5,6,7], however, the role played by altered opioid transmission in PD (compensatory vs. pathological) has not been resolved [1,8,9]. In response to DA depletion, striatal PPE-A mRNA and enkephalin peptide levels are upregulated [5,10,11,12,13], while striatal PPE-B mRNA and dynorphin are downregulated [5,12,13,14,15]. In theory, the activation of DORs by enkephalin could attenuate the hyperactivation of the indirect striatopallidal output pathway seen in PD, which inhibits movement in the Parkinsonian state. This is supported by evidence that DOR agonists have shown anti-Parkinsonian actions in preclinical studies [16,17]. On the other hand, chronic levodopa therapy is associated with elevated PPE-A levels in both preclinical models [18] and patients with LID [19]. This long-term effect on PPE-A is more consistent with a persistent adaptive role for enkephalin in LID [20]. Levels of PPE-B, on the other hand, are reversed and upregulated following chronic levodopa therapy [13,18,19]. The importance of the opioid neurotransmitter system has been further established by positron emission tomography studies which have revealed abnormalities in the expression of striatal opioid receptors in patients with LID [21]. Altogether, this suggests that increased opioid neurotransmission, especially MOR overactivity, may be associated with the pathophysiology of LID. However, our understanding of the opioid system in the context of LID has been further complicated by the failure of two pan-opioid receptor antagonists without subtype-selectivity, naltrexone and naloxone, in clinical trials [22,23].

Although treatments based on opioid transmission are not presently available, further study of opioid peptides in PD and LID has the potential to identify novel therapeutic agents and targets for these movement disorders. In particular, the glycosylation of central nervous system (CNS)-active peptides to increase their stability and CNS penetrance might provide a potent strategy for developing novel drug candidates. In our view, opioid glycopeptides offer an advantage over alkaloid-based small molecule compounds, since there is a reduced likelihood of side effects. The opioid glycopeptides are metabolized only to harmless di- and tripeptides, amino acids and sugars [24,25,26], while alkaloid-like drugs produce a cascade of toxic metabolites, with unpredictable variation between individuals.

In prior work, we demonstrated that the mixed μ/δ-activating opioid glycopeptide lactomorphin (MMP-2200) penetrated the blood-brain barrier (BBB) [27], and had behavioral effects in preclinical rodent models of PD [28] and LID [29]. Of specific interest, we demonstrated a strong interaction of an opioid peptide with the glutamatergic system using a standard model of LID. Prior preclinical studies using 6-hydroxydopamine (6-OHDA)-lesioned rats had demonstrated that the selective *N*-methyl-D-aspartate (NMDA) receptor antagonist MK-801 could reduce LID but unfortunately worsened Parkinsonism at the same time [30], specifically by the action of MK-801 on the *indirect* striatopallidal pathway [31]. We demonstrated that co-administration of MMP-2200 attenuated the pro-Parkinsonian activity of MK-801 without interfering with its anti-dyskinetic activity [29].

In the current study we investigated a novel glycopeptide BBI-11008 with selective DOR agonism. BBI-11008 is an analogue of deltorphin, which is a potent heptapeptide secreted from the skin of frogs (genus *Phyllomedusa bicolor*) [32]. The BBI-11008 receptor profile has been established by [^3^H] binding from Chinese hamster ovary (CHO) cell membranes expressing hDOR, hMOR, hKOR (δ Ki = 14 nM, μ Ki = 1100 nM, no κ binding) [33]. In that same study, the effects of BBI-11008 on acute, inflammatory and neuropathic pain, respiration and drug self-administration was investigated [33]. BBI-11008 was shown to possess broad-spectrum antinociceptive and anti-allodynic activity across a range of pain-like conditions. The profile for BBI-11008 in respiration and drug self-administration assays also suggested that BBI-11008 may have less pronounced side effects than standard MOR-agonists such as morphine or fentanyl. In the experiments described here, we systemically administered BBI-11008 and quantified its CNS penetration into the striatum of rats utilizing microdialysis and liquid chromatography-mass spectrometry (LC-MS) analysis. We then tested BBI-11008 in the standard preclinical rodent LID model [34], both alone and in combination with the NMDA receptor antagonist MK-801.

## 2. Results

### 2.1. BBI-11008 Reached High Concentrations in the Rodent Dorsolateral Striatum after Systemic Injection

**Figure 1:**
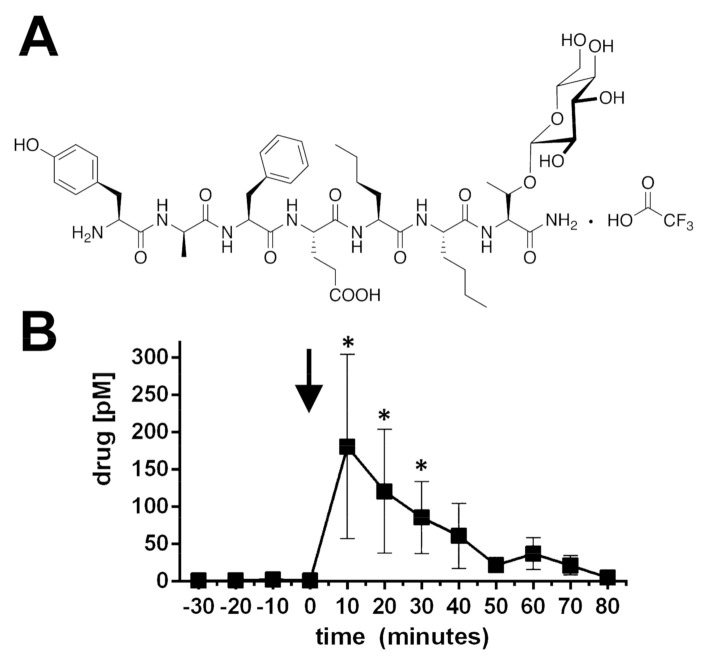
(**A**) Structure of BBI-11008. The native deltorphin sequence YaFEVVG-CONH_2_ was modified to produce the glycopeptide YaFENleNleT(β-D-Glc)-CONH_2_, where the two L-valine residues have been replaced with L-nor-leucines (Nle), and the terminal glycine has been replaced with the glucoside of L-threonine. Several related glycopeptide structures were screened for MOR and DOR agonism. (**B**) Proof of blood–brain barrier penetration of the opioid glycopeptide BBI-11008 as determined by microdialysis in the dorsolateral striatum after systemic administration. BBI-11008 (10 mg/kg, *i.p.*) rapidly reached the dorsolateral striatum as measured by in vivo microdialysis and subsequent mass spectrometric analysis in awake, freely moving rats, implanted 24 h prior to the experiments (fully recovered, wounds healed, covered by a stage that holds the probe in place). Mean concentration ± SEM is plotted against time. A high concentration of BBI-11008 was measured in the dialysate from the first time point after injection (black arrow), with >150 pM at peak level, and remained at active levels at 1 h (~37 pM). For comparison the endogenous opioid peptide leu-enkephalin measured simultaneously was determined to be >20 pM (* *p* < 0.05, *n* = 6, repeated measures ANOVA).

CNS penetration of the glycopeptide BBI-11008 (Figure 1A), a DOR agonist, was verified with microdialysis in the dorsolateral striatum (DLS) of male rats followed by LC-MS. Following *intraperitoneal* (*i.p.*) dosing with 10 mg/kg a highly significant (*p* < 0.05, *n* = 6, repeated measures ANOVA, Figure 1B) level of BBI-11008 (post hoc tests at single time points: * *p* < 0.05, *n* = 6, Figure 1B) can be measured from dialysate of the DLS. At its peak level, at 10 min post-injection, the BBI-11008 concentration in the DLS was >150 pM. The mean (±SEM) striatal BBI-11008 concentration at 60 min post-injection was reduced, 37.10 pM (±21.37), but still present at a higher level compared to that of endogenous leu-enkephalin (<20 pM), which was measured at the same time for comparative reasons. BBI-11008 did not change the endogenous levels of leu-enkephalin in the DLS (data not shown).

### 2.2. Verification of the 6-Hydroxydopamine-Lesion

**Figure 2:**
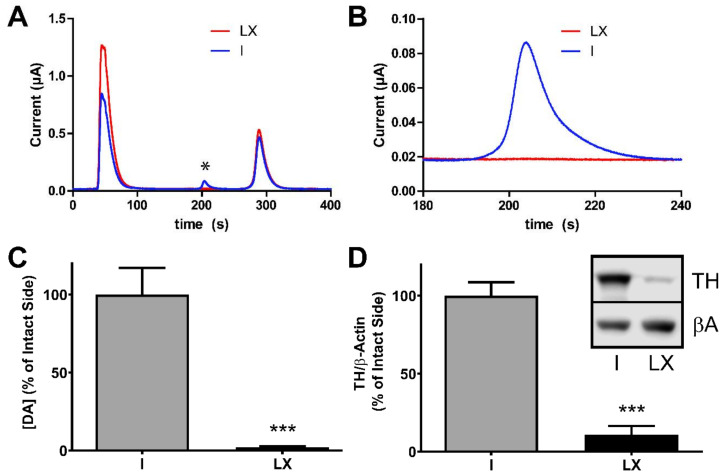
Verification of unilateral 6-OHDA lesion. (**A**–**C**) Electrochemical detection of striatal dopamine (DA) content. Representative separation of biogenic amines from tissue punches (**A**). The DA peak occurs around 200 s indicated by the *. The right panel (**B**) shows a zoom in of the DA peak. The peak area was integrated for analysis. The lesioned hemisphere (LX = lesioned) is in red, while the control hemisphere (I = intact) is in blue. (**C**) The DA content (mean ± SEM) is reduced by >95% in the lesioned side (*** *p* < 0.001, *n* = 10, two-tailed *t*-test). (**D**) Semi-quantitative Western analysis of striatal tyrosine hydroxylase (TH) expression. TH was normalized to β-actin (βA) as internal standard, mean values ± SEM are plotted, and TH expression is reduced by ~90% in the lesioned side (*** *p* < 0.001, *n* = 10, two-tailed *t*-test) verifying the severity of the lesion. The inset shows representative example Western Blots (I = intact, LX = lesioned).

In order to identify rats for the evaluation of AIMs, the severity of the 6-OHDA-lesion was estimated using the amphetamine-induced rotation test (AIR). The net ipsiversive rotations per min (mean ± SEM) of the selected cohort was 6.3 ± 1.5 (*n* = 10), indicating a lesion size > 90%. Post-mortem, the 6-OHDA lesion was confirmed using both high pressure liquid chromatography with electrochemical detection (HPLC-EC) of striatal DA levels and semi-quantitative Western Blot analysis of tyrosine hydroxylase (TH). There was a >97% loss in DA in the lesioned compared to intact hemisphere (*** *p* < 0.001, *n* = 10, two-tailed *t*-test, Figure 2A–C). TH-immunoreactivity, quantified by Western Blot analysis, was significantly decreased by 90% in the lesioned side (*** *p* < 0.001, *n* = 10; two-tailed *t*-test, Figure 2D).

### 2.3. Establishment of Limb, Axial, Orolingual and Locomotor Abnormal Involuntary Movements

Rats identified as having a lesion, via the AIR test, were treated with a combination of L-DOPA and benserazide for 3 weeks to establish limb, axial, orolingual (LAO) and locomotor AIMs. Prior to experimental testing that was scored by an experimentally blinded investigator, each rat was tested on 3 different days in order to identify rats with established LAO and locomotor AIMs (mean ± SEM). Over the 3 days the rats selected for the experiment had LAO AIMs of 54.3 ± 6.3 and locomotor AIMs of 14.3 ± 1.7 (*n* = 10), which is considered to be a moderate level of LID in this model.

### 2.4. BBI-11008 Reduced L-DOPA-Induced Locomotor Abnormal Involuntary Movements

**Figure 3:**
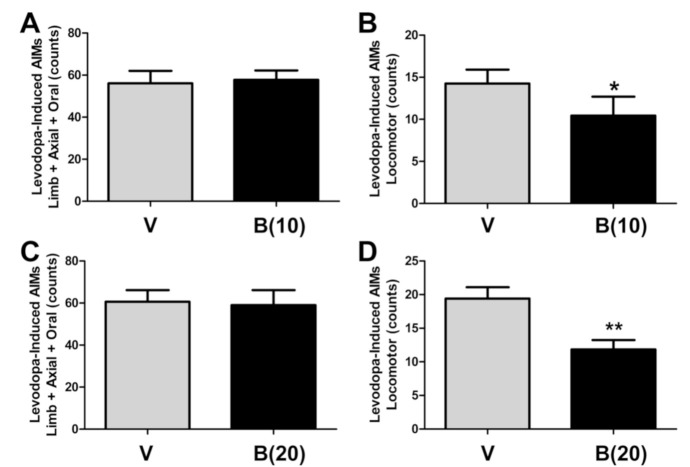
At the dose of 10 mg/kg (*i.p.*) BBI-11008 had no effect on limb, axial and oral (LAO) abnormal involuntary movements (AIMs), but significantly reduced locomotor AIMs. At this dose, BBI-11008 had also been shown to be effective in rodent models of pain. (**A**) The mean total LAO AIMs scores over 180 min are plotted and there was no difference between vehicle (gray bar) and drug (black bar) condition (mean AIMs count ± SEM; *n* = 10; paired Wilcoxon signed-rank test). **(B)** The mean total locomotor AIMs scores over 180 min were plotted and there was a significant 30% reduction from vehicle (gray bar) in the drug (black bar) condition (mean AIMs count ± SEM, * *p* < 0.05, *n* = 10, paired Wilcoxon signed-rank test). At the dose of 20 mg/kg (*i.p.*) BBI-11008 had no effect on LAO AIMs, but reduced locomotor AIMs. (**C**) The mean total LAO AIMs scores over 180 min were plotted and there was no difference between vehicle (gray bar) and drug (black bar) condition (mean AIMs count ± SEM, *n* = 10, paired Wilcoxon signed-rank test). (**D**) The mean total locomotor AIMs scores over 180 min were plotted and there was a significant 50% reduction from vehicle (gray bar) in the drug (black bar) condition (mean AIMs count ± SEM, ** *p* < 0.01, *n* = 10, paired Wilcoxon signed-rank test).

While having no effect on L-DOPA-induced LAO AIMs (Figure 3A,C) at either dose, BBI-11008 (*i.p.*), significantly reduced L-DOPA-induced locomotor AIMs at both 10 mg/kg (* *p* < 0.05, *n* = 10, paired Wilcoxon signed-rank test, Figure 3B) and 20 mg/kg (** *p* < 0.01, *n* = 10, paired Wilcoxon signed-rank test, Figure 3D) doses. 

### 2.5. BBI-11008 Did Not Affect Either the Anti-Dyskinetic Nor the Pro-Parkinsonian Activity of MK-801

**Figure 4:**
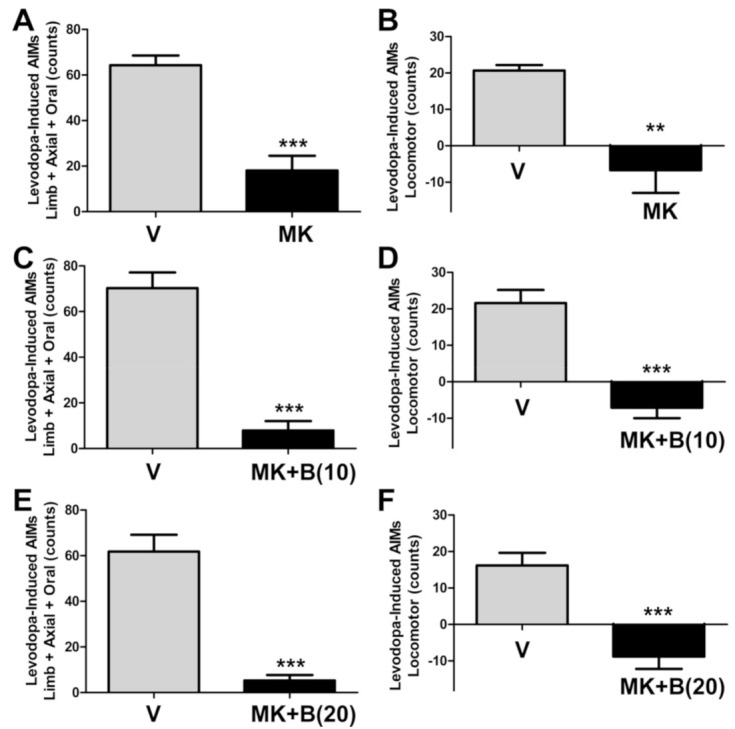
The DOR-specific opioid glycopeptide BBI-11008 did affect neither the anti-dyskinetic nor the pro-Parkinsonian activity of MK-801. (**A**) MK-801 (0.3 mg/kg) efficiently blocked LAO AIMs. Co-injection of 10 mg/kg (**C**) and 20 mg/kg (**E**) BBI-11008 (*i.p.*) did not change the effect of MK-801 to reduce LAO AIMs (*p* = 0.24, Kruskal-Wallis test of MK vs. MK+B(10) and MK+B(20) groups in A, C and E). (**B**) MK-801 (0.3 mg/kg) caused ipsiversive turns, a surrogate measure for pro-Parkinsonian activity in this model. Neither 10 mg/kg (**D**) nor 20 mg/kg (**F**) BBI-11008 abolished MK-801-induced ipsiversive locomotor AIMs (*p* = 0.8, Kruskal–Wallis test of MK vs. MK+B(10) and MK+B(20) groups in B, D and F). Data in all graphs are presented as mean AIMs count ± SEM, *** *p* < 0.001, ** *p* < 0.01, *n* = 5, paired Wilcoxon signed-rank tests vs. corresponding vehicle control.

In a subset of animals (*n* = 5) we tested BBI-11008 and MK-801 together (Figure 4). As previously established, MK-801 (0.3 mg/kg; *i.p.*) does reduce LAO-AIMs (*** *p* < 0.001, *n* = 5, paired Wilcoxon signed-rank test, Figure 4A) and induces ipsiversive pro-Parkinsonian locomotor activity (** *p* < 0.01, *n* =5, paired Wilcoxon signed-rank test, Figure 4B). At both 10 mg/kg and 20 mg/kg BBI-11008 given together with the NMDA receptor antagonist MK-801 had no effect on the known strong anti-dyskinetic effect of MK-801 (*p* = 0.24, Kruskal–Wallis test, Figure 4C,E), or the known induction of the pro-Parkinsonian locomotor activity by MK-801 (*p* = 0.80, Kruskal–Wallis test, Figure 4D,F).

## 3. Discussion

The results from our study demonstrate that BBI-11008 is CNS penetrable and achieves a significant central behavioral effect after systemic administration. This adds to the recent literature that has demonstrated that glycosylation can increase the ability of peptides to penetrate the BBB and to produce centrally-mediated behaviors following *parenteral* administration (intravenous, intraperitoneal, subcutaneous) [25,28,29,33,35,36,37,38].

Using microdialysis, coupled with mass spectrometric detection, we demonstrate that a physiologically meaningful level of the DOR-specific opioid glycopeptide BBI-11008 was achieved in our area of interest, the DLS, following *i.p.* administration; at the peak level BBI-11008 (>150 pM) reached a 10-fold higher level than that of the baseline expression of endogenous leu-enkephalin, which was measured for comparison. Furthermore, BBI-11008 levels remained higher than the level of endogenous leu-enkephalin at 1 h post-administration. The CNS penetration of the parent peptide deltorphin has not been proven using this in vivo method. While BBI-11008 has a significant antinociceptive effect in the tail-withdrawal assay [33], the parent peptide deltorphin lacks this activity in mice after intravenous administration (Bilsky and Polt, personal communication), indicating less CNS availability after systemic administration. While microdialysis is invasive, it has been demonstrated that the BBB integrity is not acutely compromised by probe implantation established at the time of our measurement [27,39,40,41]. Although some controversy remains regarding the integrity of the BBB following microdialysis surgeries [42], performing experiments 24 h after probe implantation as done here is considered the most suitable method for maintaining BBB integrity. Other factors such as lowering the probe slowly, using narrow (~300 µm) concentric probes and a low flow rate (0.7 µL/min) are also important technical considerations in minimizing mechanical disruption of the BBB [40].

Our prior studies using a rodent model of LID showed that the mixed μ/δ-opioid glycopeptide MMP-2200 had a potent effect on movements related to dopaminergic hyper-stimulation following striatal DA depletion [28] and LID [29]. Taken together with the BBI-11008 data presented here, these results are consistent with the viewpoint that the upregulation of enkephalin occurs following LID as a compensatory reaction that partially restores homeostasis in the basal ganglia circuitry. We believe that the mixed MOR/DOR opioid agonist MMP-2200 and the selective DOR agonist BBI-11008 are both able to restore the proper balance between the *direct* and *indirect* pathways, by mirroring the effect of enkephalin. Importantly, we have found that these glycopeptides reduced locomotor AIMs, but not the LAO AIMs, suggesting a possible effect of providing some benefit in LID, without worsening the primary symptoms of PD. We acknowledge, however, that the predictive validity of L-DOPA-induced contralateral locomotor AIMs as a correlate of human LID is a contentious question. For example, it has been suggested that contralateral locomotor AIMs reflect the extent of unilateral DA-denervation in the 6-OHDA-lesion rat model, while LAO AIMs are a better behavioral correlate of human LID [34,43]. 

The combination of the mixed μ/δ-opioid agonist glycopeptide MMP-2200 and the NMDA receptor antagonist MK-801 was shown to have a strong anti-dyskinetic effect without inducing the pro-Parkinsonian locomotor activity seen with MK-801 alone [29]. The modulatory activity of MMP-2200 to block the MK-801-induced pro-Parkinsonian activity could be partially disrupted by the DOR-specific antagonist naltrindole [29]. Therefore, we partially attributed the mechanism of action of MMP-2200 to agonism at the DOR. Paradoxically, we now show that BBI-11008, a selective DOR agonist, was not able to block either the anti-dyskinetic or the pro-Parkinsonian effect of MK-801 in the LID model, further indicating that a critical balance of MOR- and DOR-agonism is required for this modulation. One limitation of the presented work is that only males were tested, and therefore we cannot rule out different sex-specific effects on females. 

The necessity of this dual action is supported by consideration of a number of prior studies. For example, a recent study did show that the novel molecule DPI-289, a mixed DOR agonist/MOR antagonist, was demonstrated to be anti-dyskinetic in both rodent and non-human primate LID models [44]. In addition, the mixed KOR agonist/MOR antagonist nalbuphine was shown to reduce LID in dyskinetic non-human LID primates [45]. Blockade of the MOR receptor alone, with two highly-selective MOR antagonists, ADC-02520849 and CTAP, did not reduce LID [9,46], while a MOR antagonist with a very low selectivity, ADL5510, was successful in reducing LID [47]. This is perhaps due to action at multiple sites that includes DOR activity. Consistent with the lack of effect of pure MOR antagonists, is the study of ADC-02265510, a MOR agonist which has shown anti-dyskinetic activity [9]. It is further interesting that sub-anesthetic ketamine treatment has recently been shown to be anti-dyskinetic and anti-Parkinsonian in rodent models [48,49]. While being mostly known as a NMDA receptor antagonist, ketamine binds to both MOR and DOR at a similar affinity compared to NMDA receptors [50]. The central antinociceptive properties of ketamine in the CNS are mediated by both µ- and δ-opioid receptors [51], suggesting that the anti-dyskinetic effect may also be mediated in part by the opioid system.

In summary, and when considered with the existing literature, the effects of BBI-11008 indicate that the modulation of more than one opioid receptor might be needed for a successful anti-dyskinetic strategy that engages the opioidergic system. The results of our research with both MMP-2200 and BBI-11008 clearly demonstrate meaningful penetration of glycosylated opioid peptides into the CNS. The lack of a BBI-11008 effect in reducing the pro-Parkinsonian effects of MK-801 indicates that BBI-11008 is not a therapeutic candidate for LID, yet it can be used as an experimental tool to evaluate opioid receptor-specific effects relevant to LID. In addition, this suggests that further development of synthetic opioid peptides for the treatment of LID should focus on a mixed spectrum of opioid receptor activity rather than being highly-selective for the DOR alone. The presented work further demonstrates that glycopeptide analogues possess CNS-penetration that is sufficient to achieve a central behavioral effect. Moreover, BBI-11008 with its DOR-specific activity may prove to be a potent tool in defining the necessary spectrum of activity required to therapeutically modulate basal ganglia activity in the treatment of PD and LID. This is important, since dyskinesia matters for individuals with PD, and represents a significant source of discomfort that is still not adequately treated in many cases [52].

## 4. Materials and Methods

### 4.1. Synthesis of BBI-11008

The peptides and glycopeptides were prepared in the Polt Laboratory using Fmoc-protection (fluorenylmethoxycarbonyl protecting group) and solid-phase synthesis, similar to previously published methods [53,54]. The native deltorphin sequence YaFEVVG-CONH2, discovered by Mignogna, et al. [55], was modified by the addition of a carbohydrate, and minor modification of the lipophilic valine residues (see Table 1). The glycopeptide BBI-11008, YaFENleNleT(β-D-Glc)-CONH_2_ was chosen as a lead structure.

*Resin Preparation:* Rink amide-MBHA resin (0.83 mm/g substitution, 0.361 g, 0.3 mmol) was placed in a 12 mL fritted syringe, and washed with 5 mL of dimethylformamide (DMF) on a tumbler for 2 min. The solvent was expelled, and the DMF washing was repeated a second time. 

*Fmoc Cleavage:* A mixture of 2%DBU-2%piperidine in DMF (5 mL) was added, and the syringe was tumbled for 5 min. The basic solution was first expelled, the cleavage treatment was repeated, and tumbled for 10 min. The deprotected NH_2_ resin was loaded with DMF (5 mL) and the syringe was tumbled for 2 min. The solvent was expelled, and the washing was repeated 4X with DMF. The resin was washed with N-methylpyrrolidinone (NMP) and tumbled a final time for 2 min. 

*Glycosylated Fmoc Amino Acid Attachment:* [N-(9-fluorenylmetoxycarbonyl)-L-serine-3-yl]-2,3,4,6-tetra-O-acetyl-β-O-glucopyranoside (237 mg, 0.36 mmol, 1.2 eq.) and hydroxybenzotriazole (HOBT) (49 mg, 0.36 mmol, 1.2 eq.) were placed in a vial and dissolved in 2 mL NMP. Into the solution 56 mL (0.36 mmol, 1.2 eq.) N,N′-Diisopropylcarbodiimide (DIC) was added. The mixture was shaken for 1 min and the mixture was added to the resin. The syringe was tumbled for 5 min and then it was placed to the center of the rotating plate of a commercial 1200-watt microwave oven (Emerson, MW8992SB). The oven was set at power level 1 and operated for 10 min. During this time, every 2 min the syringe was shaken manually for 10 s and returned to the microwave. The solvent was expelled from the syringe and the resin was washed once with NMP and 5X with DMF the same way as it was described above. The Fmoc protecting group and the washing protocol was repeated for each residue, as described above. 

*Fmoc amino acid coupling:* All unglycosylated amino acid couplings were accomplished with 2 equivalent amounts of DIC, HOBT and the desired amino acid in 2 mL of NMP as described above for the glucosyl serine coupling. After the final coupling, Fmoc group removal as well as DMF washing of the resin was repeated 5X with 5 mL dichloromethane (DCM) for 2 min. 

*Acetyl groups cleavage:* The DCM-wetted solid support was loaded with 5 mL of 80% H_2_NNH_2_ x H_2_O in CH_3_OH 2 × 30 min, and 1 × 1 h, followed by two washes with 5 mL of 1:1 MeOH/CH_2_Cl_2_ and six washes with 5 mL CH_2_Cl_2_ and then drying in vacuum.

*Peptide cleavage:* The dried glycopeptides were cleaved from the resin in the same fritted syringe with a 5 mL of cocktail mixture (9.0 mL of TFA, 1.0 mL of CH_2_Cl_2_, 0.25 mL of Et_3_SiH, 0.25 mL of H_2_O, and 0.05 mL of anisole) for 2 h at RT. After the cleavage was complete, the solution was expelled. Each resin was washed 2X with 2.5 mL of the cleavage cocktail, and the expelled solutions were concentrated with a N_2_ stream to produce oils of about 3 mL in volume. After concentration 10 mL of chilled (0 °C) Et_2_O was used to precipitate each glycopeptide as an off-white powder. Each glycopeptide was centrifuged, dried, re-dissolved in water, freeze-dried and purified by HPLC to provide 310 mg of the drug candidate. 

*Opioid Receptor Binding:* Opioid receptor binding assays were performed as previously described [33]. Membranes from CHO cells stably expressing either the human DOR, MOR, or KOR were used in the binding experiments. Initially, deltorphin analogues were tested at 100 nM and 1 µM for inhibition of the binding of 0.2 nM [^3^H]naltrindole, a DOR antagonist, and 0.25 nM [^3^H]DAMGO, a MOR agonist. Peptides that produced greater than 60% inhibition at of DOR at 1 µM and less than 50% inhibition of MOR binding were characterized in a 12-point inhibition binding study to determine the Ki values for the peptides at the DOR and MOR. [^3^H]U69593 at a concentration of 1 nM was used to measure binding to the KOR. Table 1 shows the receptor binding results for the deltorphin analogues. BBI-11008 was chosen for further study.

### 4.2. Animals for Microdialysis

Adult male Sprague–Dawley rats (Harlan, Indianapolis, IN, USA) weighing between 250 and 350 g were used for all experiments (*n* = 6). Rats were housed in a temperature and humidity-controlled room with 12 h light/dark cycles with food and water available ad libitum. All animals were treated as approved by the University of Michigan Unit for Laboratory Animal Medicine (Protocol: 08516; approval date: 4 April 2011) and in accordance with the National Institute of Health (NIH) Guidelines for the Care and Use of Laboratory Animals. The number of animals used, and their suffering were minimized.

### 4.3. Animals for L-DOPA-Induced Dyskinesia Model

Male Sprague-Dawley rats (250 g; Harlan, Indianapolis, IN, USA), were housed in a temperature and humidity-controlled room with 12 h light/dark cycles with food and water available ad libitum. All animals were treated as approved by the Institutional Animal Care and Use Committee at the University of Arizona (Protocol number: 10-204; amendment approval date: 20 August 2012) and in accordance with the NIH Guidelines for the Care and Use of Laboratory Animals. The number of animals used, and their suffering were minimized.

### 4.4. In Vivo Microdialysis

We performed in vivo microdialysis in the rat DLS and applied LC-MS analytical techniques to quantify brain concentrations of the deltorphin analogue BBI-11008, following systemic treatment, and of endogenous leu-enkephalin levels both before and after treatment. These experiments were performed in awake, freely moving rats, implanted with microdialysis probes in the DLS 24 h prior to the experiments. The animals were fully recovered, their wounds healed, and the probes were fixed in place with dental cement. The details of the microdialysis procedure to detect glycopeptides in the rat striatum has been described in detail in [27], with the following minor modifications: the concentric microdialysis probes were slightly larger at 3 mm, and the microdialysis probes were flushed at a lower flow rate of 0.7 µL/min.

### 4.5. Unilateral 6-Hydroxydopamine-Lesion Rat Model of Parkinson’s Disease

All surgical procedures were performed as published in our prior work unless otherwise stated [29,48,49]. In order to prevent damage to noradrenergic neurons, each rat was pretreated with desipramine hydrochloride (12.5 mg/kg, prepared in 0.9% sterile saline with 10% dimethylsulfoxide (DMSO), *i.p.*; MilliporeSigma, St. Louis, MO, USA) 30 min prior to 6-OHDA exposure. Rats were then anesthetized with isoflurane (1.5–2.0%; VetOne, Boise, ID, USA) mixed in a vaporizer (JD Medical, Phoenix, AZ, USA) with 1.5 L of oxygen per min. 6-OHDA (5.0 µg/µL, prepared in 0.9% sterile saline with 0.02% ascorbic acid; MilliporeSigma, Burlington, MA, USA) was prepared fresh every 2 h. Using a microinjector (Stoelting Quintessential Stereotaxic Injector Model 53311, Stoelting Co., Wood Dale, IL, USA) connected to a syringe (10 µL; Hamilton Co., Reno, NV, USA) and needle (26 gauge; Hamilton Co.) two microliters (10 µg per coordinate) of 6-OHDA was administered (0.5 µL per min) unilaterally at two coordinates (in mm) within the medial forebrain bundle: AP-2.8, ML-1.8, DV-8.0 and AP-4.7, ML-1.5, DV-7.9, according to the Atlas of Paxinos and Watson, 2007 [56]. The syringe was left in place for 5 min post-injection to prevent the backflow of solution. 

### 4.6. Amphetamine-Induced Rotation Test

The AIR test was performed as previously published [29,48,49]. Then, 2 weeks post-surgery, the severity of the 6-OHDA lesion was estimated with dextroamphetamine (5.0 mg/kg, *i.p.*; MilliporeSigma). Rats were placed in a plexiglass cylinder (38 cm diameter × 38 cm height) and their net ipsiversive (towards the lesion) rotations (mean ± SEM) were counted for 1 min, every 5 min, over a total period of 100 min. 

### 4.7. Induction of L-DOPA-Induced Dyskinesia in Unilateral 6-OHDA-Lesioned Rats

Rats showing ipsiversive rotations were selected and treated daily (*i.p.*) for 3 weeks with a combination of L-DOPA (7 mg/kg) and benserazide (14 mg/kg, MilliporeSigma). After priming, all subsequent L-DOPA injections and testing occurred every 3–4 days. Baseline L-DOPA-induced LAO and locomotor AIMs (mean ± SEM) were determined over 3 testing sessions, before testing of the compounds was begun. On their respective testing days vehicle or BBI-11008 (10 and 20 mg/kg, *i.p.*) were injected at the same time as L-DOPA; MK-801 (0.3 mg/kg, *i.p.*, MilliporeSigma) was given 25 min prior.

### 4.8. Behavioral Analysis in the L-DOPA-Induced Dyskinesia Rat Model

L-DOPA-induced AIMs were scored by an experimentally blinded investigator according to [57]. We used a ‘within subjects cross over design’ to have a vehicle control experiment for every drug dose tested and to reduce variability of the behavioral results. In order to quantify the severity of the AIMs, rats were observed individually in their standard cages every 20th min between 20 and 180 min after an injection of L-DOPA. As described in the literature [30,43], AIMs were classified into four subtypes: (1) limb, i.e., jerky and/or dystonic movements of the forelimb contralateral to the lesion; (2) axial, i.e., dystonic or choreiform torsion of the trunk and neck towards the side contralateral to the lesion; (3) orolingual, i.e., twitching of orofacial muscles, and bursts of empty masticatory movements with protrusion of the tongue towards the side contralateral to the lesion; (4) locomotor, i.e., increased locomotion with contralateral side bias. The latter AIM subtype does not provide a specific measure of dyskinesia [57], but rather provides a correlate of contralateral turning behavior in rodents with unilateral 6-OHDA lesions. Each of the four subtypes was scored on a severity scale from 0 to 4, where 0 = absent, 1 = present during less than half of the observation time, 2 = present for more than half of the observation time, 3 = present all the time but suppressible by external stimuli, and 4 = present all the time and not suppressible by external stimuli. LAO AIMs have been shown to be modulated in a similar way. Therefore, scores from these three AIMs subtypes were summed. The sum of (1) LAO, or (2) locomotor AIMs scores per testing session were used for statistical analyses, as done in prior work [29,48,49]. 

### 4.9. Measurement of Striatal Dopamine Content 

Rat brains were washed in chilled Tris buffer (pH 7.4, 15 mM Tris, 125 mM NaCl, 2.5 mM KCl, 2 mM CaCl_2_) for 30 s and placed in a chilled brain matrix. Coronal brain slices were collected and a 2 mm steel biopsy punch was used to sample tissue from the striatum. Samples from the left and right hemispheres of the brain were collected and immediately flash frozen on an aluminum pan at −70 °C. Samples massed at 2.5 ± 0.5 mg and were placed in 1.5 mL homogenization vials with 100 µL of 0.1 N HClO4 (aq), manually homogenized (15 strokes) using a disposable pestle and stored at −80 °C for up to 2 weeks prior to analysis. HPLC-EC was used to separate and quantify DA, as published [48,49]. A mobile phase composed of a citrate–acetate buffer at pH 4.0 was utilized with 3% *v*/*v* of each methanol and acetonitrile. Ethylenediamine-tetraacetic acid was added at 50 ppm and n-octylsulfonic acid was added at 125 ppm to act as an ion pairing agent. A Phenomenex Synergi 2.5 um Fusion-RP 100 Angstrom, LC Column 50 × 2 mm was used on a Amersham Biosciences AKTA HPLC with a flow rate of 0.3 mL/min. Electrochemical detection was performed at a 2 mm glassy carbon electrode held at 750 mV vs. Ag/AgCl. Prior to analysis each sample was spiked with 50 ppb caffeic acid as internal standard. A total of 50 µL of sample were injected onto the HPLC system. External calibration standards were prepared in 0.1 N HClO4 with 10% *v/v* MeOH at concentrations ranging from 2 ppm to 20 ppb for DA.

### 4.10. Western Analysis of Striatal Tyrosine Hydroxylase Content

After the tissue punch, described above, the remaining striata from the left and right hemisphere was dissected, immediately flash frozen in liquid nitrogen, and stored at −80 °C. Total protein was prepared by homogenizing (BBX24-CE Bullet Blender homogenizer, Next Advance, Inc., Averill Park, NY, USA) striatal tissue in ice-cold phosphate buffered saline containing 1% Triton X-100, 0.1% sodium dodecyl sulfate, a protease inhibitor cocktail (1:100; P8340, MilliporeSigma) and a phosphatase inhibitor cocktail tablet (Roche, Basel, Switzerland). Lysate was then centrifuged for 30 min at 14,000× *g*. Supernatant was collected and added to 2× Laemmli Buffer in a 1:1 ratio and heat-treated for 5 min at 100 °C. 30 µg of protein was loaded for each sample. After being run and transferred, blots were first probed with a TH antibody (1:2000; AB152, MilliporeSigma) and then stripped and probed for β-Actin (1:10,000; A2228. MilliporeSigma), which was used as an internal loading control. A goat, anti-rabbit (1:5000; #7074, Cell Signaling Technology, Danvers, MA, USA) and horse, anti-mouse (1:5000; #7076, Cell Signaling Technology) horseradish peroxidase conjugated secondary antibody was used against each primary, respectfully. Blots were then detected using chemiluminescence (Amersham ECL Western Blotting Detection Reagent, Cytiva, Marlborough, MA, USA). Images of each blot was taken on a G:Box XR5 Chemi system (Syngene, Frederick, MD, USA) and analyzed with Image Studio Lite (LI-COR, Lincoln, NE, USA).

### 4.11. Data Analysis

Statistical analysis was performed using GraphPad Prism 5.1 software (GraphPad Software, Inc., La Jolla, CA, USA), Origin 9.0, and Microsoft Excel 2013. Repeated measures ANOVA was used to evaluate statistical differences over the course of the microdialysis experiment; Fisher Least significant difference post-hoc tests were used to evaluate the statistical difference between baseline and post-injection of BBI-11008 at different time points. For the DA and TH analyses two-tailed t-tests of the raw data before normalization was conducted. Non-parametric paired Wilcoxon signed-rank test were used to compare the effect of treatment on LAO- and locomotor AIMs vs. the matching vehicle injection, and for multiple comparisons non-parametric Kruskal-Wallis tests were utilized. The null hypothesis was rejected when *p* < 0.05. 

## Figures and Tables

**Table 1:** Opioid activity of the screened deltorphin analogues.

Sequence		% Inhibition at DOR 100 nM, 1 µM	% Inhibition at MOR 100 nM, 1 µM	Ki DOR (nM)	Ki MOR (nM)	Ki KOR (nM)
DPDPE		72, 84	23, 62	4.3	180	
H_2_N-YaFDVVG-S(β-D-Glc)-G	LSZ-27	51, 67	6, 48	33	570	
H_2_N-YmFHLM-S(β-D-Glc)	BBI-11001	72, 84	63, 89			
H_2_N-YaFHLA-S(β-D-Glc)	BBI-11002	55, 77	82, 97			
H_2_N-YmFHLMT-(β-D-Glc)	BBI-11003	75, 83	57, 89			
H_2_N-YaFHLAT-(β-D-Glc)	BBI-11004	61, 76	88, 98			
H_2_N-YaFE-Nva-Nva-S-(β-D-Glc)	BBI-11005	65, 80	11, 44	20	710	5.3%
H_2_N-YaFE-Nle-Nle-S-(β-D-Glc)	BBI-11006	66, 81	7, 42	11	820	38%
H_2_N-YaFE-Nva-Nva-T-(β-D-Glc)	BBI-11007	64, 80	13, 36	16	1300	0%
H_2_N-YaFE-Nle-Nle-T-(β-D-Glc)	BBI-11008	74, 84	7, 37	14	1100	2.9%
H_2_N-YaFE-Nle-Nle-T	BBI-11009			11	510	3.7%
H_2_N-YaFEII-T-(β-D-Glc)	BBI-11014			80	2600	36%
H_2_N-YaFEVV-S-(β-D-Glc)	BBI-11015			320	1500	0%
H_2_N-YaFE-Nle-Nle-S-(βGlc-βGal)	BBI-11021			17	1500	23%
H_2_N-YaFEVV-S-(βGlc-βGal)	BBI-11022			3600	1000	71%
H_2_N-YaFE-Nle-Nle-S-(α-D-Man)	BBI-11023			8.2	840	4.7%
H_2_N-YaFE-Nva-Nva-S-(α-D-Man)	BBI-11024			13	990	0%
H_2_N-YaFE-Nle-Nle-S-(βGlc-βGlc)	BBI-11025			13	1200	46%
H_2_N-YaFE-Ile-Ile-S-(βGlc-βGlc)	BBI-11026			61	3000	2.7%

Nva = *nor-*Valine, Nle = *nor-*Leucine, Glc = Glucose, Gal = Galactose, Man = Mannose.

## Abbreviations

[^3^H]	Tritium
6-OHDA	6-hydroxydopamine
AIMs	Abnormal involuntary movements
ANOVA	Analysis of variance
AIR	Amphetamine-induced rotation
BBB	Blood-brain barrier
BBI-11008	glycosylated δ-opioid receptor agonist
βA	Beta Actin
CHO	Chinese hamster ovary
CNS	Central nervous system
DA	Dopamine
DMF	Dimethylformamide
DMSO	dimethylsulfoxide
DCM	Dichloromethane
DIC	N,N′-Diisopropylcarbodiimide
DOR	δ opioid receptor
DLS	Dorsolateral striatum
Fmoc	fluorenylmethoxycarbonyl
GABA	gamma-aminobutyric acid
Gal	Galactose
Glc	Glucose
HPLC-EC	High performance liquid chromatography with electrochemical detection
HOBT	Hydroxybenzotriazole
HRP	Horseradish peroxidase
I	Intact hemisphere
*i.p.*	Intraperitoneal
LAO	Limb, Axial, and Orolingual
LC-MS	Liquid chromatography with mass spectrometry detection
L-DOPA	Levodopa, L-3,4-dihydroxyphenylalanine
LID	L-DOPA-Induced Dyskinesia
KOR	κ Opioid Receptor
LX	Lesioned hemisphere
Man	Mannose
MSNs	Medium spiny neurons
MK-801	Dizocilpine, *N*-methyl-D-aspartate receptor antagonist
MMP-2200	Lactomorphin, glycosylated mixed μ/δ-opioid receptor agonist
MOR	μ opioid receptor
NIH	National Institute of Health
NIDA	National Institute on Drug Abuse
NMDA	*N*-methyl-D-aspartate
NMP	*N*-methylpyrrolidinone
Nle	*Nor*-Leucine
Nva	*Nor*-Valine
PD	Parkinson’s disease
PPE-A, Penk	Preproenkephalin-A
PPE-B, Pdyn	Preproenkephalin-B
TH	Tyrosine Hydroxylase
